# Sex differences in flea infections among rodent hosts: is there a male bias?

**DOI:** 10.1007/s00436-014-4231-z

**Published:** 2014-11-21

**Authors:** Krzysztof Kowalski, Michał Bogdziewicz, Urszula Eichert, Leszek Rychlik

**Affiliations:** Department of Systematic Zoology, Institute of Environmental Biology, Faculty of Biology, Adam Mickiewicz University, Umultowska 89, 61-614 Poznań, Poland

**Keywords:** Sex-biased parasitism, Flea abundance, Body mass, *Apodemus agrarius*, *Apodemus flavicollis*, *Myodes glareolus*

## Abstract

Recognizing patterns of parasite distribution among wildlife hosts is of major importance due to growing risk of transmission of zoonotic diseases to humans. Thus, sex-dependent parasite distribution in higher vertebrates is extensively studied, and males are often found more parasitized than females. Male-biased parasitism may be the result of weaker immunocompetence of male hosts owing to the immunosuppressive effect of androgens. Moreover, larger hosts (males) may demonstrate higher parasite infestation levels than smaller individuals (females), as they constitute a better nutritional resource for parasites and provide them with a greater variety of niches. In the present work, we investigated sex-dependent patterns of flea distribution among three common rodent species (*Apodemus agrarius*, *Apodemus flavicollis*, and *Myodes glareolus*). We hypothesized that males have a higher flea infestation than females. We confirm male-biased parasitism in *A. agrarius* and *M. glareolus*, but not in *A. flavicollis*. Additionally, flea infestation increased with body mass in *A. agrarius*, but not in *A. flavicollis* and *M. glareolus*. The detected differences in parasite distribution among sexes are probably the result of immunosuppressive effects of androgens and spatial behavior of males.

## Introduction

Wild rodents are known to be reservoir hosts for many pathogens, which can be transmitted to other animals including humans (Varma and Page 1966; Pawelczyk et al. 2004; Karbowiak et al. 2005a; Klimpel et al. 2007). The most important ectoparasites infesting rodents are fleas and ticks (Pawelczyk et al. 2004) which by transmission of numerous pathogens (e.g., *Borrelia* ssp., *Babesia* ssp., *Trypanosoma* ssp., *Leishmania* ssp.) can cause serious diseases (Pawelczyk et al. 2004; Karbowiak et al. 2005b; Millán et al. 2014). The importance of rodents as a reservoir of zoonotic diseases is further stressed by their ubiquity, common distribution, and disturbance tolerance (Kryštufek and Griffiths 2002; Bogdziewicz and Zwolak 2014). Due to the high risk of infection in humans, pathogens and their hosts have become a hot spot in public interest (Morand and Poulin 1998; Klimpel et al. 2007; Dziemian et al. 2014).

To allow an accurate description of the pathogen transmission and parasite infestation, extensive research has been conducted on the distribution of fleas in host populations (Morand et al. 2004, Klimpel et al. 2007; Khokhlova et al. 2009a; Krasnov et al. 2011a,b; Kiffner et al. 2013; Kowalski et al. 2014). Evidence has shown that males of higher vertebrates are infested by more parasites than females (Khokhlova et al. 2011; Krasnov et al. 2005, 2011a; Kiffner et al. 2014). In polygynous mating systems, intra-sexual competition favors males that invest more in secondary sexual traits and grow larger. Because of energy constraints, those investments are hypothesized to be done at the expense of immunity (Rolff 2002). Another associated explanation is that in many species males have larger home ranges and often move over longer distances than females (Shenbrot et al. 1997; Stanko et al. 2002, Haapakoski and Ylönen 2010; Krasnov et al. 2011a,b), what may result in encountering more parasitic infectious stages. Furthermore, home range overlaps are usually significantly larger for males than for females; this facilitates the horizontal transmission of directly transmitted ectoparasites between males during encounters (Krasnov et al. 2005, 2011a, b).

On the other hand, females of many rodent species usually solitarily nurse their offspring (Clutton-Brock 1991, Lonstein and De Vries 2000, Girard et al. 2002) which facilitates the vertical transmission of ectoparasites between them and juveniles (Yamamura 1993; Ebert and Herre 1996). Moreover, fleas lay eggs in the host nest and therefore female nest may be of higher nutritional value than solitary male nest (Gorrell and Schulte-Hostedde 2008). Indeed, female-biased parasitism has been reported by Krasnov et al. (2005) in several rodent species in South Africa and by Patterson et al. (2008) in 16 neotropical bat species. Thus, despite common, male-biased parasitism is not a universal rule (Kiffner et al. 2013).

It has also been hypothesized that larger hosts (that is males in many/most rodents species) provide a greater variety of niches for parasites and thus can sustain a higher number of parasites (Morand and Poulin 1998; Balashov et al. 2007; Lindenfors et al. 2007). Furthermore, according to the well-fed host hypothesis, they usually represent better nutritional resource for parasites (Christe et al. 2003). Smaller individuals may be also forced to groom more because of the greater surface to volume ratio as the same density of parasites would remove a greater proportion of the hosts’ blood (Mooring et al. 2004; Harrison et al. 2010). However, there is still no consensus which of the above mentioned mechanisms is the predominant driver of male-biased parasitism (Gorrell and Schulte-Hostedde 2008; Harrison et al. 2010; Kiffner et al. 2014).

In the present work, we aimed to test the hypothesis that males of three common rodent species (*Apodemus agrarius*, *Apodemus flavicollis*, and *Myodes glareolus*) have a higher flea infestation than females. To better understand the underlying factors influencing rates, we additionally tested for weight differences between sexes and for differences in infestation in relation to body mass.

## Materials and methods

### Study area and trapping procedure

We live-trapped small mammals in the summer of 2010 and 2011 at four distinct localities across Poland (Słowiński National Park, Gorzowska Forest, Konin lakes area, and Bieszczady Mountains). In each, we established two to eight trapping plots and conducted trapping for 2 to 10 consecutive days. Detailed description of study sites and trapping procedure is provided in Kowalski et al. (2014). Captured animals were determined to species and weighed. We recorded also their age, sex, and reproductive activity and marked them individually by ear tagging or cutting a small patch of fur. Then mammals were placed in a canvas bag for 2–3 min to collect fleas (e.g., Haas and Walton 1973; Paramasvaran et al. 2009; Zuo et al. 2011; Kowalski et al. 2014). Collected fleas were placed in a vial with alcohol and mammals were released at the place of capture.

### Data analysis

We tested for differences in body mass between sexes using GLM with log-transformed individual body mass as a response variable and sex and species interaction as fixed factors. To analyze between-sex differences in flea infestation, we used generalized linear mixed models (GLMMs) implemented via the R package lme4 (Bates et al. 2013) and run separate analysis for each investigated species: *A. agrarius* (model 1), *A. flavicollis* (model 2), and *M. glareolus* (model 3). All models were fitted using Poisson family error terms and log link function with trapping site included as a random effect to control for spatial variability in infestation (Kiffner et al. 2013, 2014; Kowalski et al. 2014). In model 2, we detected overdispersion and therefore included additional random effect with unique identifier for each observation (i.e., for each individual sampled); this procedure allows for correction of standard errors and is identical to using overdispersed Poisson model (Zwolak et al. 2013). In each model, we used sex as fixed factor (male vs. female) and individual body mass as a covariate. All models were conducted using data obtained only from adult individuals (only first capture). Moreover, all pregnant and lactating females were removed from these analyses because of confounding effects of pregnancy on body mass. All statistical tests were carried out using R (R Core Team 2014).

## Results

We collected fleas from 69 individuals of *A. agrarius*, 152 of *A. flavicollis*, and 105 of *M. glareolus*. In sum, we collected 488 fleas individuals (70, 198, and 220, from *A. agrarius*, *A. flavicollis*, and *M. glareolus*, respectively) belonging to 11 species. Thirty-four *A. agrarius*, 79 *A. flavicollis*, and 36 *M. glareolus* did not carry any fleas. Maximum flea loads were 6 in *A. agrarius*, 14 in *A. flavicollis*, and 13 in *M. glareolus*.

**Fig. 1:**
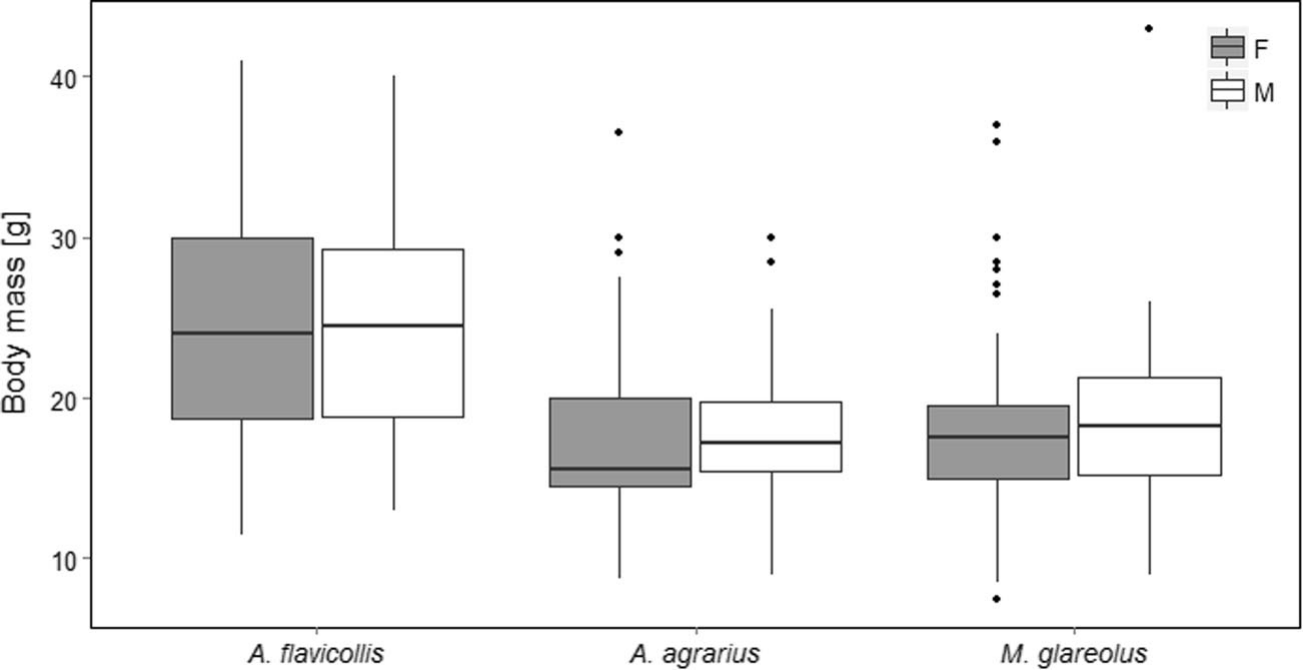
Body mass of studied species (*A. flavicollis*, *A. agrarius*, *Myodes glareolus*). Females (*F*) are shown with grey and males (*M*) with *white boxes. Boxes* denote 25th, 50th, and 75th percentiles; *whiskers* denote the furthest data points within 1.5 interquartile range

**Fig. 2:**
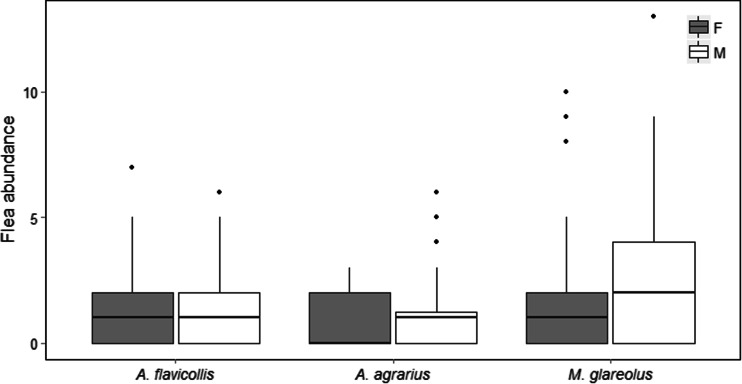
Flea abundance on males and females of three studied species (*A. flavicollis*, *A. agrarius*, *Myodes glareolus*). Females (*F*) are shown with grey and males (*M*) with *white boxes. Boxes* denote 25th, 50th and 75th percentiles; *whiskers* denote the furthest data points within 1.5 interquartile range

Males and females did not differ in body mass in our dataset (sex by species effect: *χ* ^2^ = 0.08, *df* = 2, *p* = 1.31, *p* = 0.96; Fig. 1). In *A. agrarius* and *M. glareolus*, fleas were more abundant on males than females (*A. agrarius*: *χ* ^2^ = 7.81, *df* = 1, *p* = 0.005, *M. glareolus*: *χ* ^2^ = 8.75, *df* = 1, *p* = 0.003; Fig. 2). We found no difference in parasite load between sexes in *A. flavicollis* (*χ* ^2^ = 0.16, *df* = 1, *p* = 0.68). Infestation increased with body mass in *A. agrarius* (*z*_1, 25_ = 4.07, *p* < 0.001) but not in *A. flavicollis* (*z*_1, 104_ = −0.45, *p* = 0.64) nor in *M. glareolus* (*z*_1, 33_ = −0.01, *p* = 0.98).

## Discussion

Our results confirm male-biased parasitism in *A. agrarius* and *M. glareolus*. However, parasite load did not differ between sexes in *A. flavicollis*. The detected differences in parasite distribution among sexes are probably the result of immunosuppressive effects of androgens rather than bigger size of males as body mass of sexes did not differ in our dataset (Hillegass et al. 2008; Khokhlova et al. 2009b; Kiffner et al. 2014). Moreover, flea infestation increased with body mass only in *A. agrarius*, but not in *A. flavicollis* nor in *M. glareolus*. Therefore, we can suppose that other factors than body mass, like differences in spatial behavior, movement patterns, and lower immunocompetence of males, may be responsible for found differences (Balashov et al. 2007; Hillegass et al. 2008; Krasnov et al. 2012).

Two main hypotheses regarding male-biased parasitism have been proposed (Khokhlova et al. 2010). The first hypothesis explains male-biased parasitism by the lower immunocompetence of male hosts owing to the immunosuppressive effect of androgens (Zuk and McKean 1996; Harrison et al. 2010; Khokhlova et al. 2011; Krasnov et al. 2011a). Moreover, males may invest less energy in immune responses than females because males engage in intrasexual competition and courtship (Sheridan et al. 2000; Harrison et al. 2010).

The second, not mutually exclusive, hypothesis explains this phenomenon by the higher mobility of males, which facilitates encounters with parasites (e.g., Brown et al. 1994; Khokhlova et al. 2009a; Krasnov et al. 2011a, b, 2012). Males of many rodent species have larger home ranges than females and often move over longer distances than females (Shenbrot et al. 1997; Stanko et al. 2002; Haapakoski and Ylönen 2010; Krasnov et al. 2011a, b). Furthermore, home range overlaps are usually much higher in males than females, which facilitate the horizontal transmission of ectoparasites between males during encounters (Krasnov et al. 2011a, b).

Our data corroborate results obtained by Kiffner et al. (2014), who had reported male-biased parasitism in *A. agrarius* and *M. glareolus*. However, contrary to our results, they found effect of body mass on parasite infestation level in *M. glareolus*. Morand et al. (2004) and Krasnov et al. (2011b) also found a male-biased parasitism in *A. agrarius*, *A. flavicollis*, and *M. glareolus*. However, Krasnov et al. (2011b) found higher flea load in male hosts only in winter.

As larger hosts provide a greater variety of niches for parasites and they can sustain higher number of parasites (Morand and Poulin 1998; Balashov et al. 2007), body mass can determine parasite infestation in hosts of different taxa (Stanko et al. 2002; Kiffner et al. 2013, 2014). In addition, according to the well-fed host hypothesis, larger animals should be more parasitized, as they represent a better nutritional resource (Hawlena et al. 2005; Gorrell and Schulte-Hostedde 2008). Gorrell and Schulte-Hostedde (2008) found that flea infestation was weakly correlated with body size in females of red squirrels, but not in males. In contrary, Harrison et al. (2010) suggest that male-biased parasitism in *Apodemus sylvaticus* is driven by differences in body size between sexes.

As we found the influence of host body mass on parasite load only in one out of three studied species, we suppose that other factors than body mass are responsible for found differences. Generally, males of all these three rodent species have larger home ranges and move over longer distances than females (Viitala and Hoffmeyer 1985; Vukićević-Radić et al. 2006). However, we found male-biased parasitism only in *A. agrarius* and *M. glareolus*, not in *A. flavicollis*. Presumably, because of lower immunocompetence males represent a more suitable patch for parasites than females (Zuk and McKean 1996; Harrison et al. 2010; Khokhlova et al. 2011; Krasnov et al. 2011a).

Mice of the genus *Apodemus* are the most widespread small mammals in the temperate zone of the Palearctic region (Ondriková et al. 2010). *A. agrarius* and *A. flavicollis* are very common small rodents in Poland and usually co-occur with other rodent species (e.g., *M. glareolus*). These three species show some preferences towards human settlements and agricultural areas (Ondriková et al. 2010). As pathogens can be transmitted from wild rodents to pets and humans (Karbowiak et al. 2005a; Klimpel et al. 2007), interactions between parasites and their hosts should be a hot spot in public interest. A better understanding of the mechanisms of parasitic infestation in wild animals and the transmission of pathogens to other animals including humans is necessary in an effective prevention of zoonotic diseases. Especially, data on the local prevalence and abundance of parasites of different taxa constitute a basic knowledge to raise public awareness and take a control of parasite infestation in pets and humans (Knaus et al. 2014).
